# Differences in in vitro microglial accumulation of the energy metabolism tracers [^18^F]FDG and [^18^F]BCPP-EF during LPS- and IL4 stimulation

**DOI:** 10.1038/s41598-021-92436-0

**Published:** 2021-06-24

**Authors:** Chie Suzuki, Sarina Han, Gandhervin Kesavamoorthy, Mutsumi Kosugi, Kaori Araki, Norihiro Harada, Masakatsu Kanazawa, Hideo Tsukada, Yasuhiro Magata, Yasuomi Ouchi

**Affiliations:** 1 Department of Molecular Imaging, Preeminent Medical Photonics Education and Research Center, Hamamatsu University School of Medicine, Hamamatsu, Japan; 2 Department of Biofunctional Imaging, Preeminent Medical Photonics Education & Research Center, Hamamatsu University School of Medicine, 1-20-1 Handayama, Higashi-ku, Hamamatsu, 431-3192 Japan; 3 Central Research Laboratory, Hamamatsu Photonics K.K., Hamamatsu, Japan

**Keywords:** Neurodegenerative diseases, Microglia

## Abstract

The positron emission tomography probes 2-deoxy-2-[^18^F]fluoro-D-glucose ([^18^F]FDG) and 2-*tert*-butyl-4-chloro-5-{6-[2-(2-[^18^F]fluoroethoxy)-ethoxy]-pyridin-3-ylmethoxy}-2H-pyridazin-3-one ([^18^F]BCPP-EF) are designed to evaluate glycolysis and oxidative phosphorylation, respectively, and are both used to estimate neuronal activity. However, previous studies have shown a discrepancy in these probes’ accumulation in the compromised region, possibly due to the presence of activated microglia acting like deleterious or neuroprotective phenotypes. Hence, we evaluated lipopolysaccharide (LPS)- and interleukin 4 (IL4)-stimulated microglial uptake of [^14^C]2DG and [^18^F]BCPP-EF to give a new insight into the hypothesis that different uptake of [^18^F]FDG and [^18^F]BCPP-EF can be ascribed to the different metabolic pathways activated during microglial activation. LPS or IL4 stimulation increased the proinflammatory or anti-inflammatory marker gene expression in microglial cells. In LPS-stimulated cells, [^14^C]2DG uptake and glycolysis related gene expression were elevated, and [^18^F]BCPP-EF uptake was reduced. In IL4-stimulated cells, [^18^F]BCPP-EF uptake was increased, and [^14^C]2DG uptake was decreased. The expression of genes involved in glycolysis and mitochondrial complex I subunits was not changed by IL4 stimulation. The uptake of [^14^C]2DG and [^18^F]BCPP-EF differs in LPS- and IL4-stimulated polarized microglial cells. The present results suggest that the in vivo accumulation of metabolic tracers [^18^F]FDG and [^18^F]BCPP-EF can be influenced by the different aspects of neuroinflammation.

## Introduction

Brain positron emission tomography (PET) is a powerful tool for elucidating the pathophysiology of neurodegenerative diseases in vivo^1,2^ to allow differential diagnosis of these diseases^3,4^. Currently, 2-deoxy-2-[^18^F]fluoro-D-glucose ([^18^F]FDG), an ^18^F-labeled glucose analog, is widely used as a PET tracer for assessing neural activity based on the fact that the accumulation of [^18^F]FDG reflects glycolytic activity^5,6^. Indeed, a reduction in [^18^F]FDG accumulation is observed in the atrophic region in the brains of elderly individuals^7^ and in the precuneus/posterior cingulate, lateral parietal and frontal cortices in patients with Alzheimer’s disease (AD)^8^ in parallel with the progression of AD pathology^9^. This suggests that reduced [^18^F]FDG accumulation relates to neural hypoactivity and/or neuronal loss. In contrast, previous animal studies of acute ischemic insult have shown higher [^18^F]FDG accumulation in hemodynamically compromised regions where the number of neuronal cells is reduced^10–12^. In-depth histopathological examination has revealed that a large amount of microglia gather around the ischemic core, where [^18^F]FDG accumulation is elevated^10–12^. Chronic neurodegenerative diseases are also accompanied by persistent neuroinflammation^13^. A metabolic radiotracer [^18^F]FDG can evaluate neuronal energy metabolism as well, which has been shown to decrease in the brains of chronic neurodegenerative diseases due to the overwhelming neuronal loss. The degree of [^18^F]FDG uptake in the tissue depends on the balance of activities of neurons and glial cells, and also on the conditions of acute or chronic insults. Thus, [^18^F]FDG should be re-evaluated to determine how it specifically accumulates in the brain region affected by acute injury in the presence of different types of neuroinflammation.

In vivo energy metabolism can also be evaluated with our newly developed PET tracer, 2-*tert*-butyl-4-chloro-5-{6-[2-(2-[^18^F]fluoroethoxy)-ethoxy]-pyridin-3-ylmethoxy}-2H-pyridazin-3-one ([^18^F]BCPP-EF)^14,15^, which binds to the rotenone binding site on mitochondrial complex I (MC-I)^11,14,16^, the rate-limiting enzyme of mitochondrial oxidative phosphorylation (OXPHOS)^17^. In contrast to [^18^F]FDG, which reflects glycolysis (especially anaerobic), [^18^F]BCPP-EF is considered an indicator of mitochondrial activity, reflecting oxidative metabolism. In our previous study, [^18^F]BCPP-EF accumulation in the compromised brain region was reduced, whereas the uptake of [^18^F]FDG was increased, suggesting that [^18^F]BCPP-EF might be a good indicator of neuronal damage or insensitivity to inflammatory glial activity^11,12^. However, the time course of proinflammatory and anti-inflammatory effects of microglia has been reported to be different following acute brain injury^18^, and the energy metabolism pathway activity is shown to change according to microglial activation states as follows: the glucose consumption rate is increased in LPS and interferon γ (INFγ)-stimulated microglia and decreased in IL4 stimulated microglia^19^, while the oxygen consumption rate (OCR), an OXPHOS index, is decreased in IFNγ- and LPS + IFNγ-stimulated microglia and increased in IL4-stimulated microglia^20,21^. Thus, microglial [^18^F]FDG and [^18^F]BCPP-EF accumulation could be determined by the microglial activation states according to the changes in energy metabolism activity. However, the effects of neuroinflammation on the brain accumulation of [^18^F]FDG and [^18^F]BCPP-EF was not been able to be identified, because neurons were mixed with glial cells in in vivo experiments such as our previous study.

Here, the aim of this study was to investigate whether the in vivo metabolic tracers [^18^F]FDG and [^18^F]BCPP-EF differ in their accumulations in differentially activated microglia. To specify the cellular accumulation of radiotracers in such microglia independently of neuron and the other glial cells, we used chemically-induced activated microglia in an in vitro experimental setting instead of in vivo settings where neuron and glial cells coexist.

## Results

### Expression levels of proinflammatory and anti-inflammatory marker genes in LPS- or IL4-stimulated microglial cells

**Figure 1:**
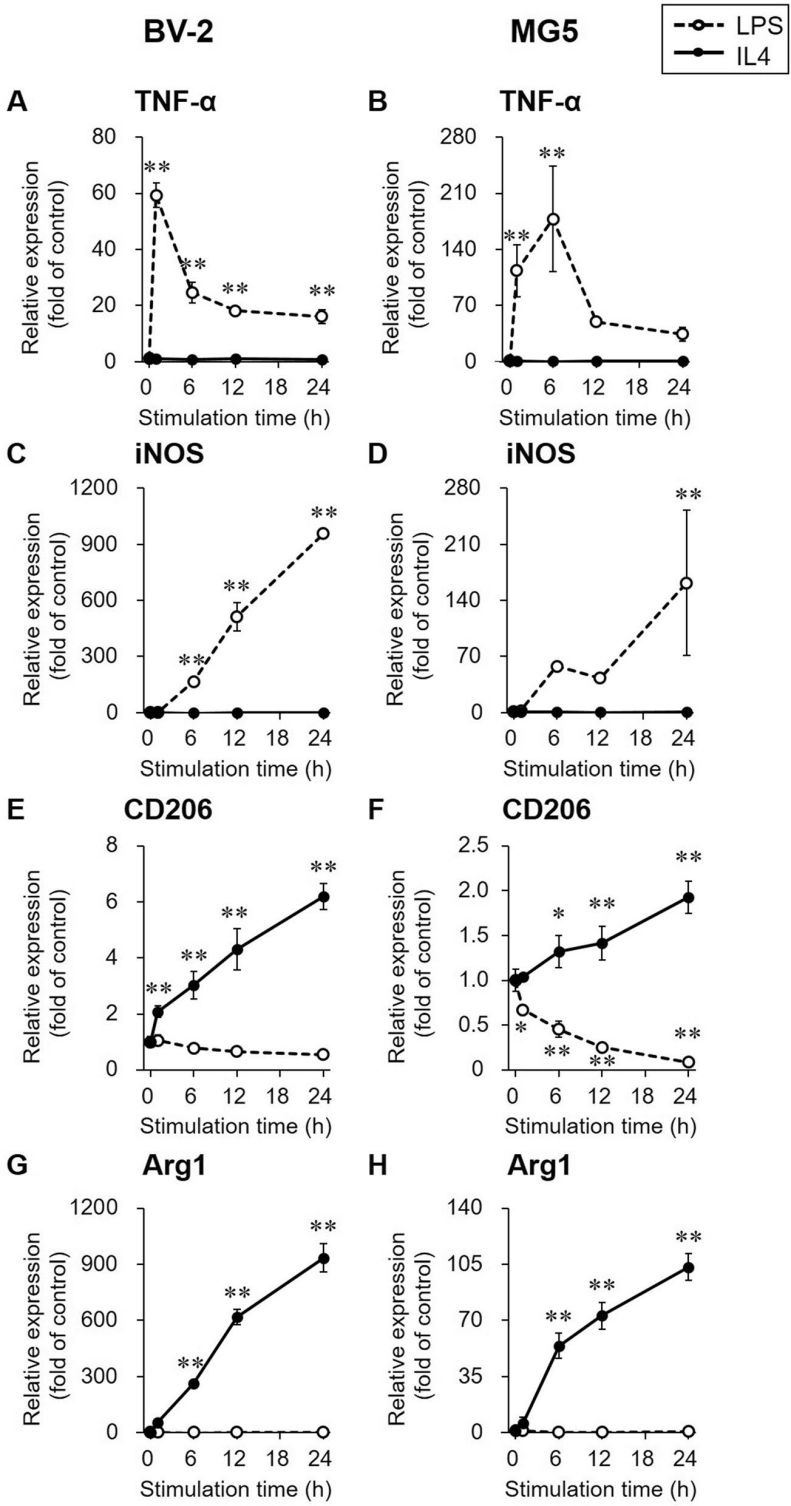
Gene expression of pro- and anti-inflammatory markers. Relative gene expression levels of the proinflammatory markers tumor necrosis factor-α (TNF-α, **A** and **B**) and inducible nitric oxide synthase (iNOS, **C** and **D**) and the anti-inflammatory marker genes CD206 (**E** and **F**) and arginase 1 (Arg1, **G** and **H**) were determined by RT-PCR in LPS (1000 ng/mL, open circle and dashed line)- or IL4 (60 ng/mL, closed circle and solid line)-stimulated BV-2 and MG5 cells. The results are presented as the fold change in expression relative to the control group (0 h stimulation) and the means ± SDs of three samples. Statistical differences were analyzed by ANOVA followed by Dunnett's multiple comparison test; *p < 0.05, **p < 0.01 compared with the control.

The gene expression of proinflammatory and anti-inflammatory markers in LPS- or IL4-stimulated BV-2 and MG5 cells is shown in Fig. 1. The mRNA levels of tumor necrosis factor-α (TNF-α) in LPS-stimulated cells were the highest at 1 h (59.3 ± 4.4-fold) in BV-2 cells and 6 h (178 ± 66-fold) in MG5 cells and then declined over time (Fig. 1A,B). The highest expression levels of inducible nitric oxide synthase (iNOS) were observed in cells after 24 h of LPS stimulation (958 ± 15-fold in BV-2 cells; 162 ± 91-fold in MG5 cells; Fig. 1C,D). A significant increase in the mRNA levels of CD206 was observed in IL4-stimulated BV-2 cells compared with control cells at 1 h (2.08 ± 0.20-fold, *p* < 0.01) and in IL4-stimulated MG5 cells compared with control cells at 6 h (1.32 ± 0.18-fold, *p* < 0.05) (Fig. 1E,F). Arginase 1 (Arg1) expression levels were also significantly increased at 6 h (262 ± sevenfold in BV-2 cells, *p* < 0.01; 54.1 ± 7.8-fold in MG5 cells, *p* < 0.01) and at later time points in IL4-stimulated cells compared with control cells (Fig. 1G,H). TNF-α and iNOS expression levels in IL4-stimulated cells (Fig. 1A–D) and Arg1 expression levels in LPS-stimulated cells (Fig. 1G,H) were not significantly different from those in control cells (*p* > 0.05). CD206 expression levels were decreased in LPS-stimulated MG5 cells from 1 h (0.67 ± 0.05-fold, *p* < 0.05; Fig. 1F).

### Glycolysis-related gene expression in LPS- or IL4-stimulated microglial cells

**Figure 2:**
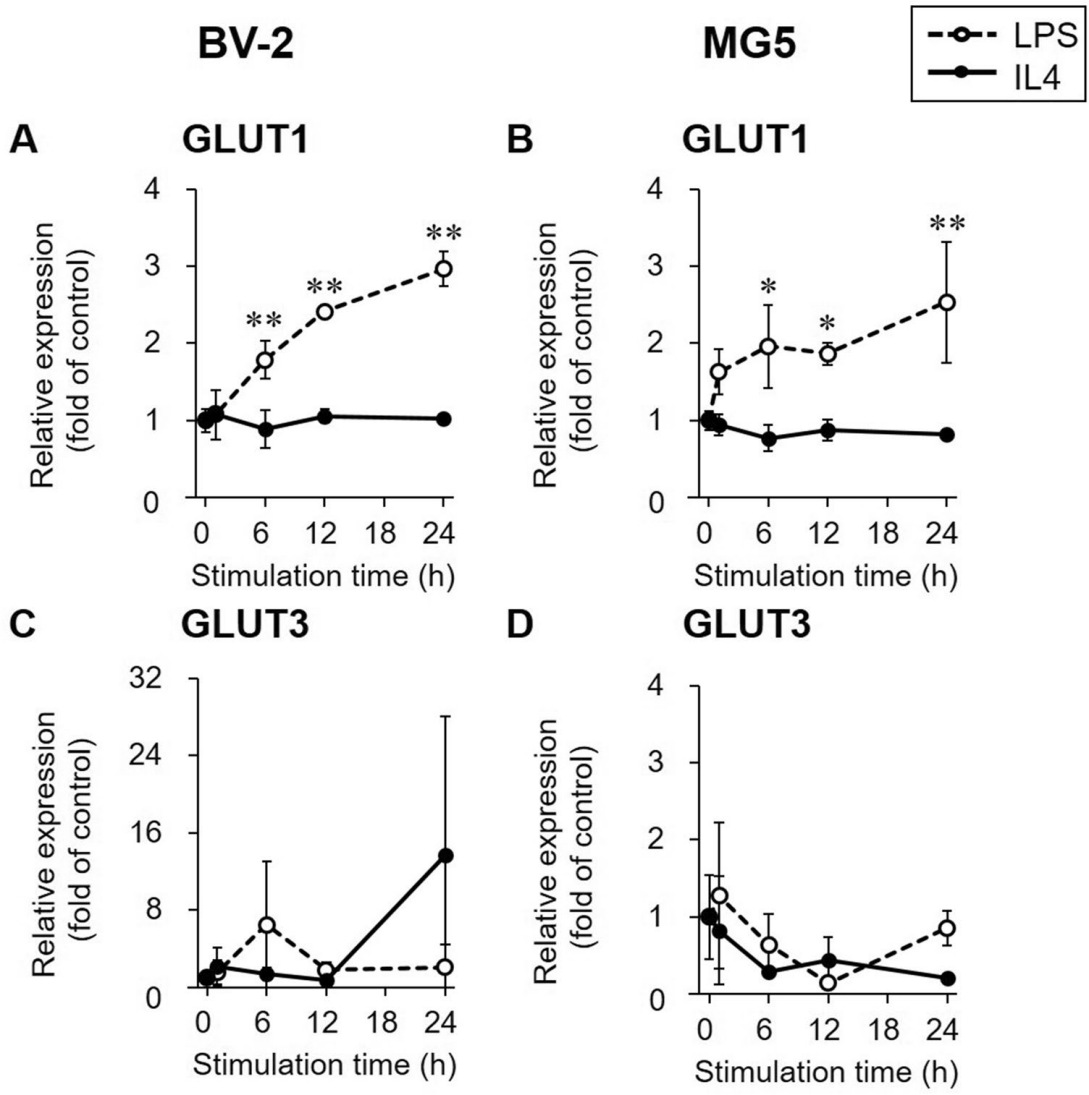
Gene expression of glucose transporters (GLUTs). Relative gene expression levels of GLUT1 (**A** and **B**) and GLUT3 (**C** and **D**) were determined by RT-PCR in LPS (1000 ng/mL, open circle and dashed line)- or IL4 (60 ng/mL, closed circle and solid line)-stimulated BV-2 and MG5 cells. The results are presented as the fold change in expression relative to the control group (0 h stimulation) and the means ± SDs of three samples. Statistical differences were analyzed by ANOVA followed by Dunnett's multiple comparison test; *p < 0.05, **p < 0.01 compared with the control.

The expression levels of glucose transporter (GLUT) 1 and 3 in LPS- or IL4-stimulated BV-2 and MG5 cells are shown in Fig. 2. GLUT1 mRNA levels increased with time in LPS-stimulated cells and did not change in IL4-stimulated cells (Fig. 2A,B). On the other hand, GLUT3 mRNA levels were not changed in either LPS- or IL4-stimulated BV-2 and MG5 cells compared with control cells (*p* > 0.05; Fig. 2C,D).

**Figure 3:**
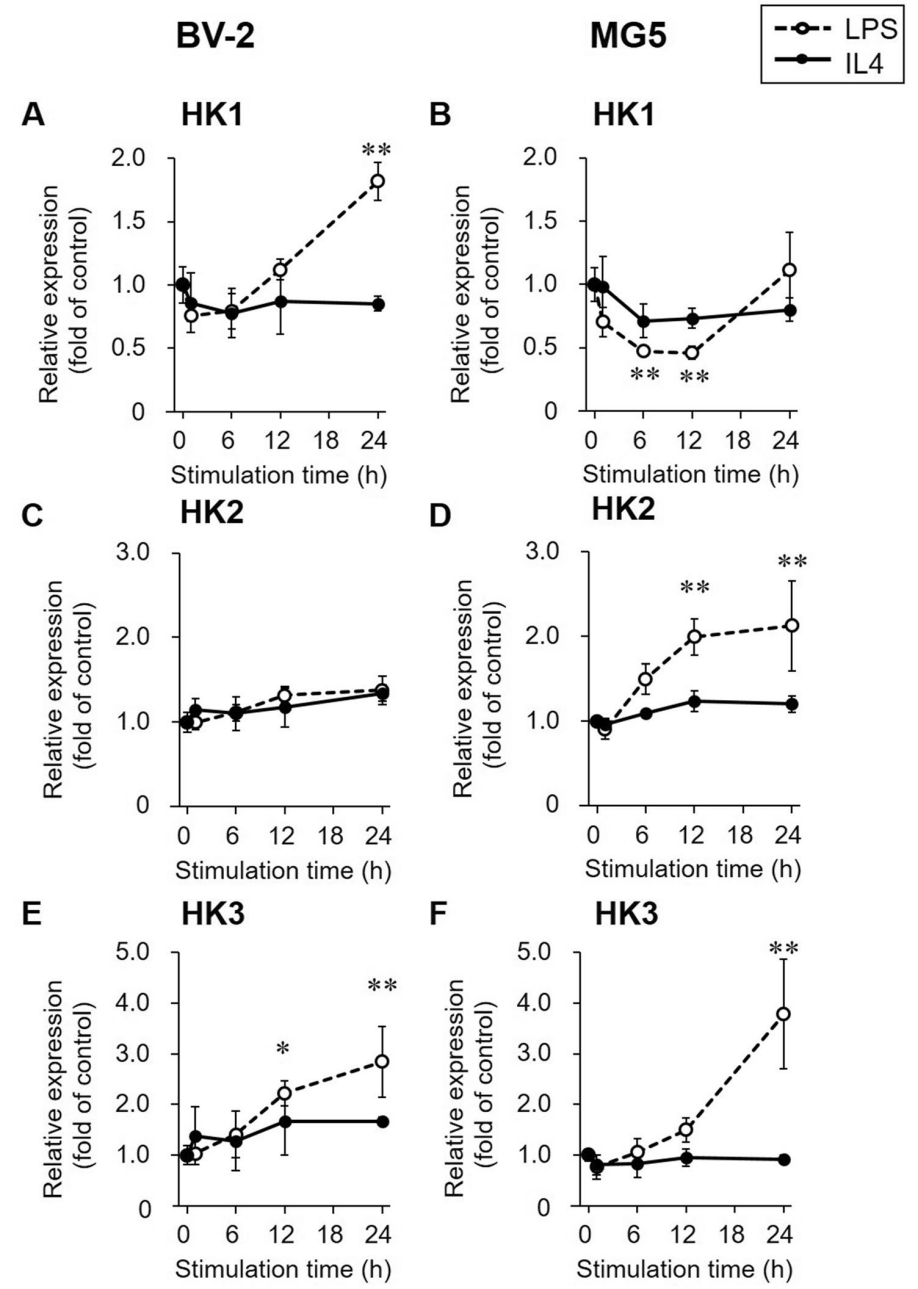
Gene expression of hexokinases (HKs). Relative gene expression levels of HK1 (**A** and **B**), HK2 (**C** and **D**), and HK3 (**E** and **F**) were determined by RT-PCR in LPS (1000 ng/mL, open circle and dashed line)- or IL4 (60 ng/mL, closed circle and solid line)-stimulated BV-2 and MG5 cells. The results are presented as the fold change relative to the control group (0 h stimulation) and the means ± SDs of three samples. Statistical differences were analyzed by ANOVA followed by Dunnett's multiple comparison test; *p < 0.05, **p < 0.01 compared with the control.

The expression levels of hexokinase (HK) in LPS- or IL4-stimulated BV-2 and MG5 cells are shown in Fig. 3. Higher expression levels of HK1 (1.82 ± 0.15-fold, *p* < 0.01) and HK3 (2.84 ± 0.69-fold, *p* < 0.01) were observed in LPS-stimulated BV-2 cells compared with control cells at 24 h, while HK2 expression levels were not changed over 24 h (Fig. 3A,C,E). HK2 (2.13 ± 0.53-fold, *p* < 0.01) and HK3 (3.78 ± 1.08-fold, *p* < 0.01) expression levels but not HK1 expression levels were increased in MG5 cells stimulated with LPS for 24 h compared with control cells (Fig. 3B,D,F). HK1, 2, and 3 expression levels were not significantly changed by IL4 stimulation in either BV-2 or MG5 cells (*p* > 0.05, Fig. 3A–F).

### MC-I subunit gene expression in LPS- or IL4-stimulated microglial cells

**Figure 4:**
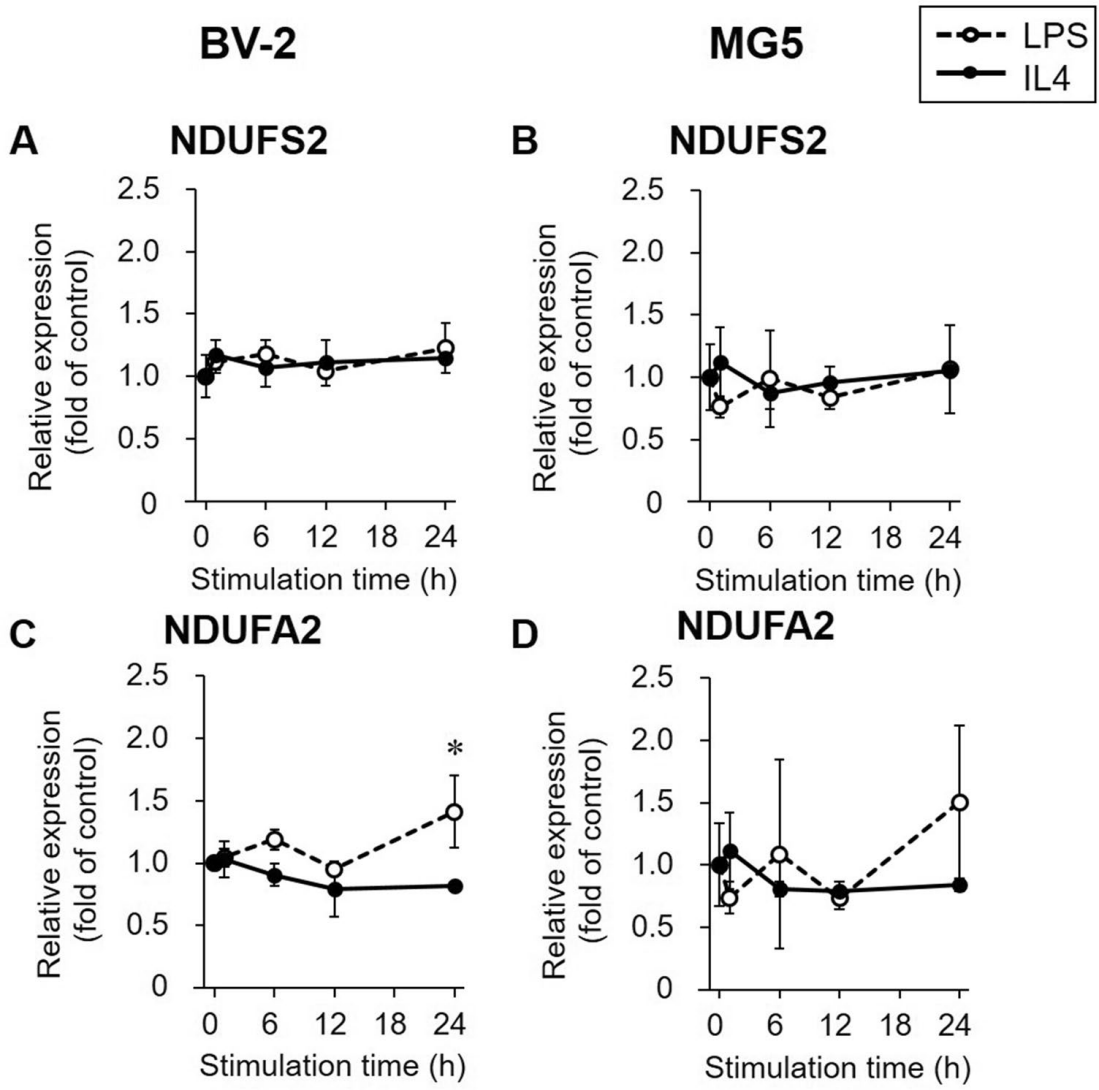
Gene expression of mitochondrial complex I subunits. Relative gene expression levels of mitochondrial complex I subunits NADH: ubiquinone oxidoreductase core subunit S2 (NDUFS2, **A** and **B**) and NADH: ubiquinone oxidoreductase subunit A2 (NDUFA2, **C** and **D**) were determined by RT-PCR in LPS (1000 ng/mL, open circle and dashed line)- or IL4 (60 ng/mL, closed circle and solid line)-stimulated BV-2 and MG5 cells. The results are presented as the fold change relative to the control group (0 h stimulation) and the means ± SDs of three samples. Statistical differences were analyzed by ANOVA followed by Dunnett's multiple comparison test; *p < 0.05 compared with the control.

The expression levels of MC-I subunits (NDUFS2, NADH:ubiquinone oxidoreductase core subunit S2; and NDUFA2, NADH:ubiquinone oxidoreductase subunit A2) in LPS- or IL4-stimulated BV-2 and MG5 cells are shown in Fig. 4. The expression levels of NDUFS2 and NDUFA2 were not markedly changed in LPS- or IL4-stimulated BV-2 and MG5 cells, although there was a slight increase in the expression of NDUFA2 in BV-2 cells stimulated with LPS for 24 h.

### Radiotracer uptake in LPS- or IL4-stimulated microglial cells

**Figure 5:**
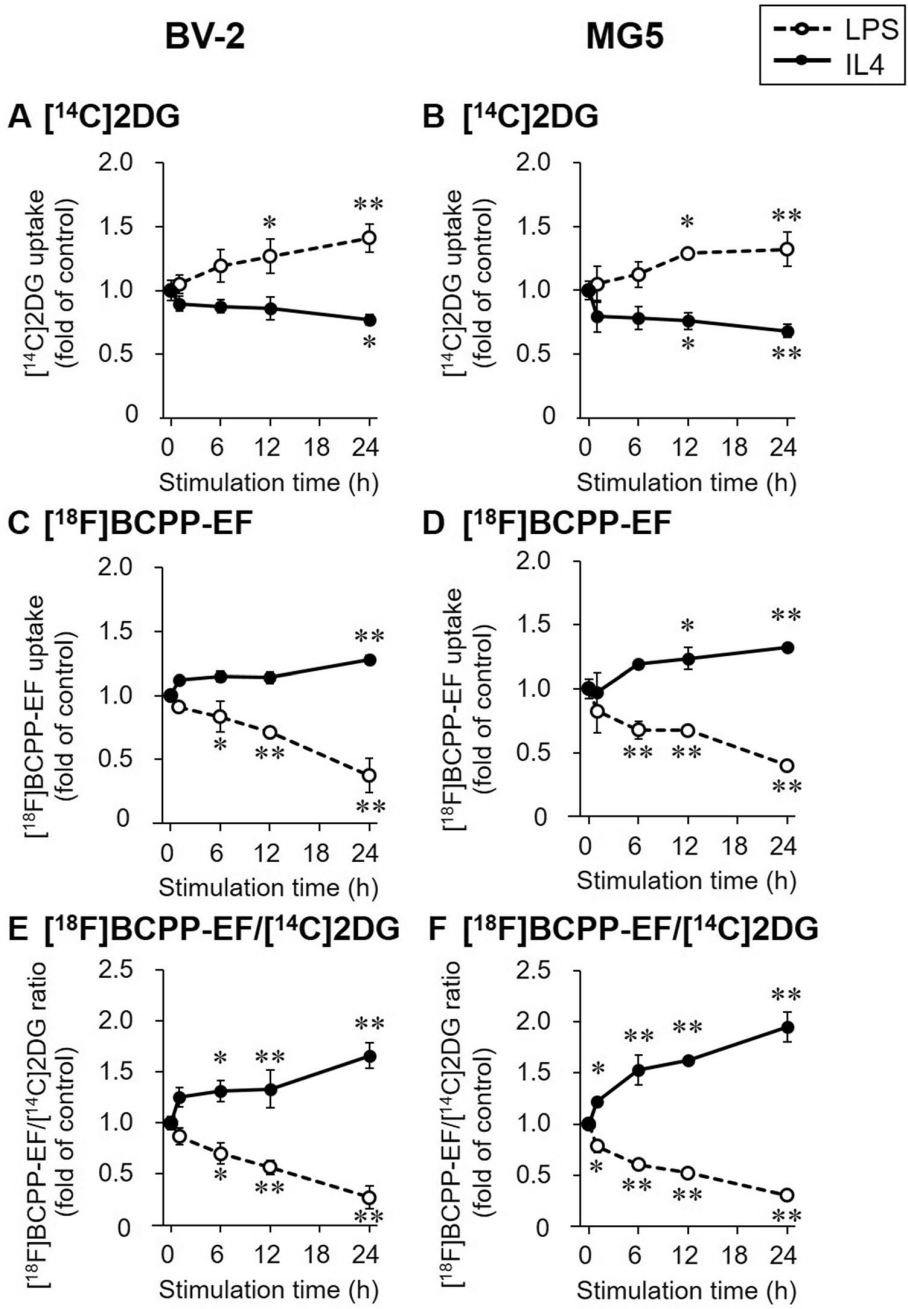
[^14^C]2DG and [^18^F]BCPP-EF uptake profiles. Cellular uptake of [^14^C]2DG (**A** and **B**) and [^18^F]BCPP-EF (**C** and **D**) was determined in LPS (1000 ng/mL, open circle and dashed line)- or IL4 (60 ng/mL, closed circle and solid line)-stimulated BV-2 and MG5 cells, and the ratio of [^18^F]BCPP-EF uptake to [^14^C]2DG uptake ([^18^F]BCPP-EF/[^14^C]2DG ratio, **E** and **F**) was calculated. The results are presented as the fold change in uptake or the uptake ratio relative to the control group (0 h stimulation) and the means ± SDs of three samples. Statistical differences were analyzed by ANOVA followed by Dunnett's multiple comparison test; *p < 0.05, **p < 0.01 compared with the control.

The specific uptake of [^14^C(U)]2-Deoxy-D-glucose ([^14^C]2DG) showed a good correlation with that of [^18^F]FDG in MG5 cells (y = 5.62x − 58.6, R^2^ = 0.97, *p* < 0.01; Suppl. Figure S1). The effects of blocking with excess of unlabeled 2DG and rotenone on [^14^C]2DG and [^18^F]BCPP-EF accumulation were shown in Suppl. Figure S2. [^14^C]2DG uptake in LPS- or IL4-stimulated BV-2 and MG5 cells is shown in Fig. 5A,B. In LPS-stimulated microglial cells, [^14^C]2DG uptake increased with time and was significantly increased compared with that in control cells at 12 h (1.27 ± 0.13-fold in BV-2 cells, *p* < 0.05; 1.29 ± 0.02-fold in MG5 cells, *p* < 0.05) and 24 h (1.41 ± 0.1-fold in BV-2 cells, *p* < 0.01; 1.32 ± 0.13-fold in MG5 cells, *p* < 0.01). In IL4-stimulated microglial cells, [^14^C]2DG uptake decreased with time and was significantly decreased compared with that in control cells at 12 h (0.760 ± 0.065-fold, *p* < 0.05) in MG5 cells and 24 h in both BV-2 and MG5 cells (0.769 ± 0.043-fold in BV-2 cells, *p* < 0.05; 0.682 ± 0.053-fold in MG5 cells, *p* < 0.01).

Figure 5C,D shows [^18^F]BCPP-EF uptake in LPS- or IL4-stimulated BV-2 and MG5 cells. [^18^F]BCPP-EF uptake was decreased with time in LPS-stimulated BV-2 and MG5 cells. Higher [^18^F]BCPP-EF uptake levels were observed in IL4-stimulated cells after 12 h in MG5 cells (1.23 ± 0.09-fold, *p* < 0.05) and 24 h in both BV-2 and MG5 cells (1.28 ± 0.03-fold in BV-2 cells, *p* < 0.01; 1.32 ± 0.03-fold in MG5 cells, *p* < 0.01).

The uptake ratio of [^18^F]BCPP-EF to [^14^C]2DG in the same cells is shown in Fig. 5E,F. The [^18^F]BCPP-EF/[^14^C]2DG uptake ratio in LPS-stimulated cells decreased with time over 24 h, and the lowest ratio was observed at 24 h (0.270 ± 0.113-fold in BV-2 cells, *p* < 0.01; 0.304 ± 0.023-fold in MG5 cells, *p* < 0.01). In contrast, a time-dependent increase in the [^18^F]BCPP-EF/[^14^C]2DG uptake ratio was observed in IL4-stimulated cells. The [^18^F]BCPP-EF/[^14^C]2DG uptake ratio in IL4-stimulated cells was higher than that in control cells at 6, 12, and 24 h for BV-2 cells (*p* < 0.05 at 6 h; *p* < 0.01 at 12 and 24 h) and at all time points tested for MG5 cells (*p* < 0.05 at 1 h; *p* < 0.01 at 6, 12, and 24 h).

## Discussion

The present in vitro study of cultured microglial cells showed that the accumulation of the in vivo metabolic tracers [^18^F]FDG and [^18^F]BCPP-EF may depend on differences in the neuroinflammatory state (proinflammatory or anti-inflammatory) in accordance with differences in the activation of energy metabolism.

While these PET tracers used in brain research are designed to evaluate energy metabolism in the living brain, a variety of intrinsic cells in the brain, such as neurons and glia, share common energy metabolism pathways, resulting in no discrimination of the degree of utilization of PET tracers among these cells. Hence, an in vitro cell culture experiment was chosen as an ideal method to determine whether the energy metabolism patterns evaluated by the metabolic tracers [^18^F]FDG and [^18^F]BCPP-EF differ in different neuroinflammatory activated states. In the present study, we used LPS to induce proinflammatory activated states and IL4 to induce anti-inflammatory activated states. We used two types of microglial cells, i.e., BV-2 cells, which were immortalized using v-raf/v-myc retrovirus^22^, and MG5 cells, which were derived from a p53-deficient mouse^23^, to exclude the effects of the immortalization procedure on energy metabolism profiles. To confirm the characteristics of the activated microglial cells, we measured changes in the expression of widely available pro- and anti-inflammatory markers through biochemical methods^24^. As shown in Fig. 1, LPS stimulation increased the expression levels of the proinflammatory markers TNF-α and iNOS from early time points after stimulation without increasing the expression of their anti-inflammatory counterparts, CD206 and Arg1. In contrast, compared with control cells, IL4-stimulated microglial cells showed increased expression levels of anti-inflammatory markers and unaltered levels of pro-inflammatory markers. These results convinced us to proceed with further experiments to evaluate radiotracer uptake in activated microglia. Prior to the radiotracer experiments, we confirmed that the uptake of two radiolabeled glucose analogs, i.e., [^14^C]2DG and [^18^F]FDG, was correlated. Since simultaneous measurement is essential for evaluating concurrent changes in metabolic responses in energy production pathways, the simultaneous administration of two different metabolic tracers was considered a prerequisite. Here, we used a combination of [^14^C]2DG and [^18^F]BCPP-EF, which enable us to separately measure these radiotracers’ uptake by distinguish radiation, instead of combination of [^18^F]FDG and [^18^F]BCPP-EF. [^14^C]2DG accumulates by the same molecular mechanisms as [^18^F]FDG: these glucose analogs are incorporated into cells via GLUT^25,26^ and phosphorylated by HKs^27^, and accumulate in a similar manner^28^. Consistently, as shown in Supplemental Figure S1, there was a good correlation between the cellular uptake of [^14^C]2DG and that of [^18^F]FDG, confirming that [^18^F]FDG could be replaced by [^14^C]2DG in our double tracer studies.

The radiotracer uptake study showed an increase in [^14^C]2DG uptake in LPS-stimulated microglial cells and a decrease in [^14^C]2DG uptake in IL4-stimulated microglial cells over time (Fig. 5A,B). To investigate whether the change in [^14^C]2DG uptake was in parallel with the concomitant change in the expression of glycolysis-related genes in polarized microglial cells, we focused on changes in the gene expression of GLUTs and HKs during LPS- and IL4-stimulation (Figs. 2 and 3). We found that [^14^C]2DG uptake in LPS-stimulated microglial cells increased as the gene expression of GLUT1 and HKs was elevated. Microglial GLUT3 expression levels were not affected by LPS or IL4 stimulation, in agreement with a previous report^21^, whereas GLUT3 expression in peripheral inflammatory cells has been reported to be increased via their activation^29^. In addition, several indicators of glycolytic activity, such as glucose consumption, extracellular lactate release, and the extracellular acidification rate (ECAR), were elevated in LPS-stimulated microglial cells, as reported in previous studies using LPS-stimulated BV-2 cells^19,21,30^. These findings suggest that LPS-stimulated microglia preferentially utilize glycolytic activities, resulting in an increase in [^14^C]2DG (and [^18^F]FDG) accumulation. In contrast, [^14^C]2DG uptake was decreased in IL4-stimulated microglial cells, although the gene expression levels of GLUTs and HKs were not changed within 24 h of stimulation. This reduction in [^14^C]2DG uptake in IL4-stimulated microglial cells was in line with a previous report showing that glucose consumption and lactate release are reduced in IL4-stimulated BV-2 cells^19^. Glycolysis suppression in IL4-stimulated microglial cells might be caused by factors other than gene expression regulation, such as negative feedback allosteric regulation^31^ and/or intracellular localization of HKs^32,33^. Although the basic mechanisms of glycolytic recruitment in LPS-stimulated microglia and reduced recruitment in IL4-stimulated microglia remain to be elucidated, it is likely that proinflammatory microglia can proliferate when the glycolytic metabolic pathway is enhanced possible under anaerobic conditions, whereas metabolic activities other than glycolysis are needed for the growth of anti-inflammatory microglia.

**Figure 6:**
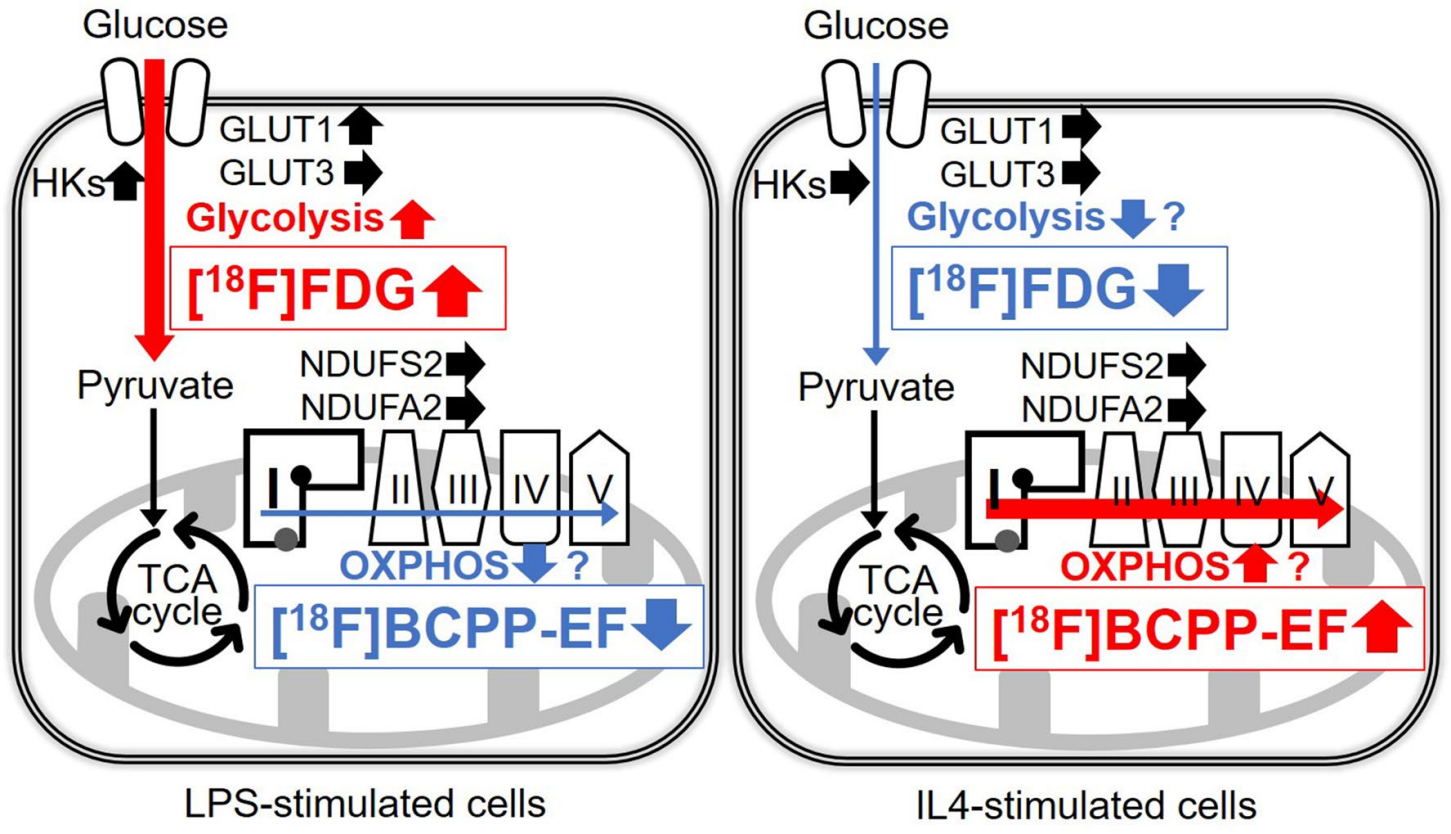
Putative changes in [^18^F]FDG and [^18^F]BCPP-EF uptake and metabolic profiles in LPS- or IL4-stimulated microglia. In LPS-stimulated microglial cells, overexpression of glycolysis-related genes (GLUT1 and HK2) was observed in the present study, and glycolysis and oxidative phosphorylation (OXPHOS) activity were previously reported to be increased and decreased, respectively. [^14^C]2DG ([^18^F]FDG) and [^18^F]BCPP-EF uptake were increased and decreased according to these changes in metabolic profiles, respectively, and the [^18^F]BCPP-EF/[^14^C]2DG ([^18^F]BCPP-EF/[^18^F]FDG) ratio was decreased. In IL4-stimulated microglial cells, glycolysis and OXPHOS activity were speculated to be decreased and increased, respectively, without a change in gene expression. [^14^C]2DG ([^18^F]FDG) and [^18^F]BCPP-EF uptake was decreased and increased according to previously reported changes in metabolic activity profiles, respectively, and the [^18^F]BCPP-EF/[^14^C]DG ([^18^F]BCPP-EF/[^18^F]FDG) ratio was increased. The red and blue arrows indicate increased and decreased expression, activity, or uptake, respectively.

The [^18^F]BCPP-EF uptake study showed reduced uptake in LPS-stimulated microglial cells and increased uptake in IL4-stimulated microglial cells with time (Fig. 5C,D). To investigate whether [^18^F]BCPP-EF uptake was associated with the gene expression levels of MC-I subunits in polarized microglia, we analyzed the expression levels of two MC-I subunit genes, NDUFS2 coding rotenone binding site^34^ and NDUFA2 coding essential subunit for MC-I assembly^35^. Our assumption was that the expression levels of these genes could affect [^18^F]BCPP-EF binding because [^18^F]BCPP-EF is thought to bind to the rotenone binding site^11,14,16^ that is encoded in NDUFA2 and because knockdown of NDUFS2 or NDUFA2 has been reported to reduce MC-I activity^35,36^. Contrary to our conjecture, it was shown that NDUFS2 and NDUFA2 gene expression levels were not markedly changed during LPS- and IL4-stimulation (Fig. 4) and that they were not correlated with [^18^F]BCPP-EF uptake levels (Figs. 4, 5C,D). Despite this, it has been reported that OXPHOS activities vary according to the activated states. Indeed, both the basal OCR and the OXPHOS reserve are reportedly decreased in LPS-stimulated BV-2 cells^21,30^, while the basal OCR is increased in IL4-stimulated cells^20^. [^18^F]BCPP-EF uptake in LPS- or IL4-stimulated microglia was in accordance with these OXPHOS activity changes. Since we evaluated gene expression levels but not MC-I activity in polarized microglia in the present study, it is too early to conclude that the different in [^18^F]BCPP-EF uptake between polarized microglia reflects only a difference in MC-I activity. However, a previous study showing that LPS impairs MC-I activity without changing MC-I protein levels in mouse liver tissue^37^ suggests that MC-I activity may be decreased due to the presence of inactive MC-I^38^ without accompanying changes in MC-I gene or protein expression levels. Taken together, these findings suggest that the mechanism of low [^18^F]BCPP-EF uptake in LPS-stimulated microglia and its high uptake in IL4-stimulated microglia might reside in the mitochondrial respiratory capacity irrespective of MC-I related gene expression or the amount of the protein, rather depending on the degree of presence of active or inactive form of MC-I (Fig. 6).

Different uptake patterns of [^14^C]2DG and [^18^F]BCPP-EF can be thought to result from the different degrees of dependance on anaerobic or aerobic metabolism in differentially polarized microglia. We estimated the degree of ATP production that shifted to anaerobic glycolysis or mitochondrial OXPHOS in LPS- or IL4-stimulated microglia by calculating the ratio of [^18^F]BCPP-EF uptake to [^14^C]2DG uptake in the same cells. It was found that the [^18^F]BCPP-EF/[^14^C]2DG uptake ratio decreased in LPS-stimulated microglia and increased in IL4-stimulated microglia (Fig. 5E,F). The decrease in [^18^F]BCPP-EF/[^14^C]2DG ratio in LPS-stimulated microglia was in line with a previous study showing that the OCR/ECAR ratio is reduced in rat primary cultured microglial cells after stimulation with LPS and IFN-γ^39^. In contrast, the [^18^F]BCPP-EF/[^14^C]2DG ratio was found to be increased in IL4-stimulated microglia in the present study, while the OCR/ECAR ratio has been reported to remain stable in rat primary cultured microglial cells after stimulation with IL4 + IL13^39^. The inconsistency between the present increase in the [^18^F]BCPP-EF/[^14^C]2DG ratio and the lack of change in the OCR/ECAR ratio in previously reported IL4 + IL13 stimulated microglia might be attributed to two things: misidentification of glycolysis activity by ECAR because of extracellular acidification irrelevant to glycolysis^40^ and the different polarization states of IL4 alone- and IL4/IL13-stimulated cells. Despite this inconsistency, it is very likely that a change in the [^18^F]BCPP-EF/[^14^C]2DG uptake ratio reflects an alteration in metabolic dependence on glycolysis or mitochondrial OXPHOS in differentially activated microglia states.

In conclusion, the present in vitro uptake experiments demonstrated that [^18^F]FDG uptake was increased in LPS-stimulated microglia and decreased in IL4-stimulated microglia and that [^18^F]BCPP-EF uptake was decreased in LPS-stimulated microglia and increased in IL4-stimulated microglia, which is in agreement with previously reported metabolic changes. The current findings indicates that in vivo microglial accumulation of [^18^F]FDG and [^18^F]BCPP-EF could be changed according to the microglial activation states, which might cause the discrepancy between neural activity and brain [^18^F]FDG and [^18^F]BCPP-EF accumulation.

## Methods

### Reagents

[^14^C]2DG was purchased from PerkinElmer (NEC720A, MA, USA). [^18^F]FDG was radiosynthesized using a [^18^F]FDG synthesizer F200 (Sumitomo Heavy Industries, Tokyo, Japan). The radiochemical purity of [^18^F]FDG was higher than 95%, as determined by thin-layer chromatography (Tec-Control chromatography system, Biodex Medical Systems, NY, USA). [^18^F]BCPP-EF was radiosynthesized as reported previously^14^. The radiochemical purity of [^18^F]BCPP-EF was 100%, as determined by high-performance liquid chromatography using L-Column2 (3 μm, 4.6 × 100 mm, CERI, Tokyo, Japan) at a flow rate of 1.5 mL/min with a mobile phase consisting of 30 mM ammonium acetate in 0.1% acetic acid aqueous solution/acetonitrile (45/55, v/v). The molar activities of [^14^C]2DG, [^18^F]FDG, and [^18^F]BCPP-EF were 0.010, 95.4, and 53.3 GBq/µmol, respectively. Lipopolysaccharides (LPS) from *Escherichia coli* O55:B5 (Sigma-Aldrich, MO, USA) and interleukin 4 (IL4, PeproTech, NJ, USA) were dissolved in Dulbecco’s phosphate-buffered saline (D-PBS (−); Nacalai Tesque, Kyoto, Japan) at concentrations of 1 mg/mL and 60 µg/mL, respectively, and stored at − 20 °C until use. Rotenone (R0090) and 2-deoxy-D-glucose (D0051) were purchased from Tokyo Chemical Industry (Tokyo, Japan).

### Cell culture and stimulation

The immortalized mouse microglial cell line BV-2^22^ was kindly provided by Prof. Ueki (Department of Integrative Anatomy, Nagoya City University) and maintained in high-glucose Dulbecco’s modified Eagle’s medium (DMEM, FUJIFILM Wako Pure Chemicals, Osaka, Japan) supplemented with 10% heat inactivated fetal bovine serum (FBS, Nichirei Bioscience, Tokyo, Japan), 100 units/mL penicillin, and 100 µg/mL streptomycin (Thermo Fisher, CA, USA). The mouse astrocyte cell line A1 (IFO50519) and mouse microglial cell line MG5 (IFO50520), which were established by Ohsawa et al*.*^23^, were obtained from the Japanese Collection of Research Bioresources Cell Bank (Osaka, Japan). A1 cells were cultured in A1 growth medium (low-glucose DMEM (Nacalai Tesque) containing 10% heat inactivated FBS, 100 units/mL penicillin, and 100 µg/mL streptomycin) to prepare A1-conditioned medium^23^. MG5 cells were maintained in a 1:1 mixture of A1-conditioned medium and A1 growth medium and were seeded in 12-well plates 28 h before the experiments at a density of 2 × 10^5^ cells/well in FBS and antibiotic-free low-glucose DMEM. BV-2 cells were seeded in 12-well plates 27 h before the experiments at the same density as MG5 cells in FBS and antibiotic-free high-glucose DMEM. The cells were then stimulated for 1, 6, 12, or 24 h before the experiments with 1000 ng/mL LPS and 60 ng/mL IL4 to induce proinflammatory and anti-inflammatory activated microglia, respectively. Unstimulated cells were prepared under the same conditions and used as controls. All cells were incubated at 37 °C in humidified 95% air and 5% CO_2_.

### Reverse transcription polymerase chain reaction (RT-PCR) analysis

Total RNA was extracted and purified from microglial cells using RNAiso Plus (Takara Bio, Shiga, Japan) according to the manufacturer’s protocol. cDNA was obtained from 500 ng of total RNA using a ReverTra Ace RT-PCR RT Master Mix kit (TOYOBO, Osaka, Japan) following the manufacturer’s instructions. cDNA (10 ng/well) was then subjected to PCR on a Thermal Cycler Dice Real Time System III instrument (Takara Bio) in a 20 µL reaction volume containing TB Green Premix Ex Taq II (Takara Bio) and 800 pmol each of the respective forward and reverse primers. The sequences of primers used are listed in Table 1. Samples were subjected to 40 cycles of amplification at 95 °C for 5 s and 60 °C for 30 s. All reactions were performed in duplicate, relative expression was quantified by the 2 − ΔΔCT method, and gene expression was normalized to the expression of β-actin as a housekeeping gene (n = 3).

**Table 1:** Sequences of primers used for RT-PCR.

Gene	Forward primer (5′–3′)	Reverse primer (5′–3′)
β-Actin	CATCCGTAAAGACCTCTATGCCAAC	ATGGAGCCACCGATCCACA
TNF-α	GTTCTATGGCCCAGACCCTCAC	GGCACCACTAGTTGGTTGTCTTTG
iNOS	GACAGCACAGAATGTTCCAG	GACTTGGACTTGCAAGTG
CD206	CTGCAAGCAGCAGAATGCTG	CAACCACTGCGTACACTCAG
Arg1	CAGAGTATGACGTGAGAGAC	GAGATGCTTCCAACTGCCAG
GLUT1	CAGGTGTTTGGCTTAGACTC	CTCGAAGCTTCTTCAGCACAC
GLUT3	CAGACGCAACTCTATGCTTC	CTCTCCAATGTACATAGGCAC
HK1	CTGGAAGACATTCGAACAGAG	GAGCAGCTCTGGAGTAATG
HK2	TCATTGTTGGCACTGGAAGC	TTGCCAGGGTTGAGAGAGAG
HK3	CTGGAATGCTTGCAGCAATTC	GTGTGGACCTCACGTATGTG
NDUFS2	CACGGAGAAGCTCATTGAG	CTATCGAATAGGCCTGTTC
NDUFA2	CTGGGGTTGCGTGAGATTC	GATCAGAATGGGCAGGTTG

### Radiotracer uptake studies

Before the assays, the cells were rinsed with 1 mL PBS (+) (D-PBS (−) containing 0.9 mM CaCl_2_ and 0.33 mM MgCl_2_). The cells were incubated with 500 µL of 0.1% *N,N*-dimethylsulfoxide in PBS (+) containing 125 kBq of [^18^F]FDG and 3.7 kBq of [^14^C]2DG or 500 kBq of [^18^F]BCPP-EF and 3.7 kBq of [^14^C]2DG for 30 min at 37 °C in humidified 95% air and 5% CO_2_ (n = 3). For the blocking studies, 10 µmol/L rotenone or 10 mmol/L unlabeled 2-deoxy-D-glucose was coincubated with the radiotracers to assess the nonspecific uptake of the radiotracers. After three rapid washes with ice-cold PBS (+), the cells were lysed with 400 µL of 0.1 M sodium hydroxide aqueous solution. A portion of each lysate (300 µL) was transferred to a scintillation vial, and ^18^F radioactivity was measured with an auto-well γ-counter (1480 Wizard 3, PerkinElmer). The protein concentration of another portion of each lysate was determined by the DC protein assay (Bio-Rad Laboratories, CA, USA). After the radioactivity of ^18^F had decreased to the background level, the samples were diluted with 3 mL scintillation cocktail (Aquasol-2, PerkinElmer), and ^14^C radioactivity was measured with a scintillation counter (Aloka LSC-6100, Hitachi, Tokyo, Japan). The net uptake of the radiotracers was normalized to the protein content. Specific uptake was calculated by subtracting nonspecific uptake, which was determined by the blocking studies, from total uptake. The ratio of [^18^F]BCPP-EF uptake to [^14^C]2DG uptake was calculated by dividing the specific uptake of [^18^F]BCPP-EF by that of [^14^C]2DG.

### Statistical analysis

All data are expressed as the mean ± standard deviation (SD) of the fold change of the control group. Statistical analyses were performed with GraphPad Prism version 5 (GraphPad Software, CA, USA). The significance of differences between the control and drug-stimulated groups was determined by one-way analysis of variance (ANOVA) followed by Dunnett's multiple comparison test. The correlation between [^18^F]FDG and [^14^C]2DG uptake was analyzed by Pearson’s correlation test. A *p* value of < 0.05 was regarded as statistically significant.

## Supplementary Information

Supplementary Figures.

## Data Availability

All data supporting this study are available within the article and its supplementary material.
