# Supplementary material for: Differences in in vitro microglial accumulation of the energy metabolism tracers [18F]FDG and [18F]BCPP-EF during LPS- and IL4 stimulation
Source: Sci Rep. 2021 Jun 24;11:13200. doi: 10.1038/s41598-021-92436-0 (PMC8225620; doi:10.1038/s41598-021-92436-0)
Supplement: Supplementary file 1 — Supplementary Figures. [file 41598_2021_92436_MOESM1_ESM.pdf]

Supplementary information for:

Differences in *in vitro* microglial accumulation of the energy metabolism tracers [^{18}F]FDG and [^{18}F]BCPP-EF during LPS- and IL4 stimulation

Chie Suzuki[1 †], Sarina Han[2 †], Gandhervin Kesavamoorthy[2], Mutsumi Kosugi[1], Kaori Araki[1], Norihiro Harada[3], Masakatsu Kanazawa[3], Hideo Tsukada[3], Yasuhiro Magata[1], Yasuomi Ouchi[2*]

[1] Department of Molecular Imaging, Preeminent Medical Photonics Education & Research Center, Hamamatsu University School of Medicine

[2] Department of Biofunctional Imaging, Preeminent Medical Photonics Education & Research Center, Hamamatsu University School of Medicine

[3] Central Research Laboratory, Hamamatsu Photonics K.K.

[†] Contributed equally

*** Corresponding author:**

Yasuomi Ouchi, Department of Biofunctional Imaging, Preeminent Medical Photonics Education & Research Center, Hamamatsu University School of Medicine, 1-20-1 Handayama, Higashi-ku, Hamamatsu 431-3192, Japan, ouchi@hama-med.ac.jp

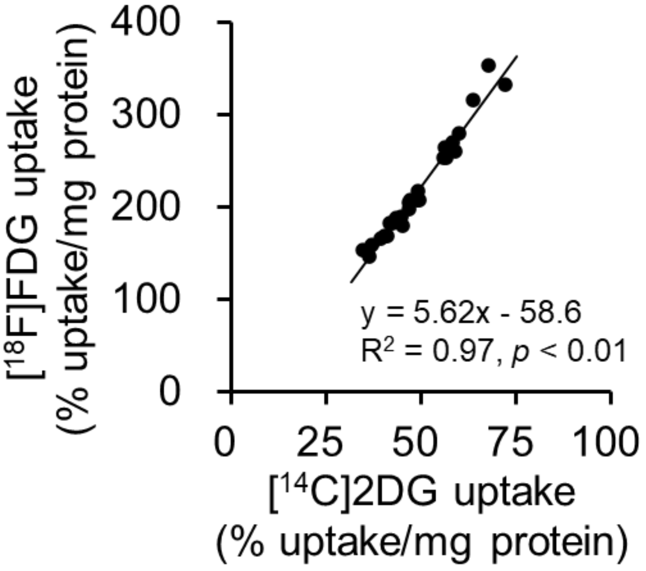

Figure S1 Correlation between [14C]2DG and [18F]FDG uptake in all untreated, LPS-stimulated and IL4-stimulated MG5 cells.

In treated cells, cellular uptake of radioligands was determined in LPS (1000 ng/mL)- or IL4 (60 ng/mL)- stimulated MG5 cells, and presented as % uptake/mg protein. Correlation between [14C]2DG and[18F]FDG uptake was analyzed by Pearson's correlation test.

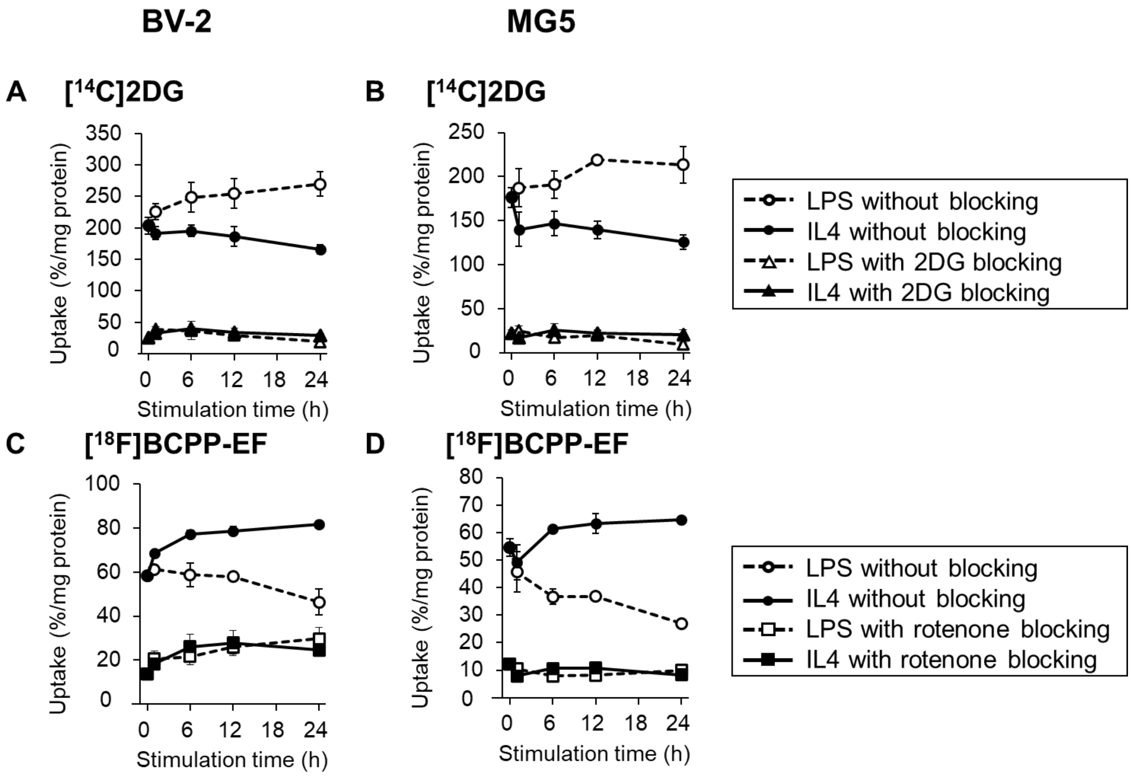

Figure S2 The effects of blocking with excess of unlabeled 2DG and rotenone on [^{14}C]2DG and [^{18}F]BCPP-EF accumulation.

Cellular uptake of [^{14}C]2DG (A and B) and [^{18}F]BCPP-EF (C and D) was measured in LPS (1000 ng/mL, open circle and dashed line)- or IL4 (60 ng/mL, closed circle and solid line)-stimulated BV-2 and MG5 cells, in absence and presence of excess of unlabeled 2DG (10 mmol/M) or rotenone (10 µmol/L). The results are presented as the percentage of uptake normalized to the protein contents and the means ± SDs of three samples.
